# Efficacy and Safety of Tripterygium Wilfordii Hook. F for Connective Tissue Disease-Associated Interstitial Lung Disease:A Systematic Review and Meta-Analysis

**DOI:** 10.3389/fphar.2021.691031

**Published:** 2021-06-10

**Authors:** Yehui Li, Wen Zhu, Hailang He, Yordan Angelov Garov, Le Bai, Li Zhang, Jing Wang, Jinghai Wang, Xianmei Zhou

**Affiliations:** ^1^ Affiliated Hospital of Nanjing University of Chinese Medicine, Nanjing, China; ^2^ Jiangsu Provincial Hospital of Chinese Medicine, Nanjing, China; ^3^ Nanjing University of Chinese Medicine, Nanjing, China

**Keywords:** tripterygium wilfordii hook F, connective tissue disease-associated interstitial lung disease, efficacy, safety, meta-analysis

## Abstract

**Background:** Tripterygium wilfordii Hook. F (TwHF), a Chinese herbal medicine used to treat CTD-ILD patients in China, has been previously found to have immunoinhibitory, antifibrotic and anti inflammatory effects. It has also shown good results in treating autoimmune and inflammatory diseases.

**Objectives:** This systematic review and meta-analysis aims to evaluate the efficacy and safety of TwHF for CTD-ILD.

**Methods:** A systematic search was performed on PubMed, Embase, Cochrane Library, Web of Science, PsycINFO, Scopus, CNKI, Wanfang, VIP, and CBM databases up to May 2021. Randomized controlled trials (RCTs) comparing TwHF plus conventional therapy versus conventional therapy alone were included. We followed the PRISMA checklist, and applied Cochrane handbook 5.1.0 and RevMan 5.3 for data analysis and quality evaluation of the included studies.

**Results:** Based on Cochrane handbook 5.1.0, nine RCTs consisting 650 patients met the inclusion/exclusion criteria and were selected for further analysis. The obtained data showed significant improvement in lung function with TwHF plus conventional treatment compared with conventional treatment (post-treatment FVC% (MD= 8.68, 95%Cl (5.10, 12.26), *p* < 0.00001), FEV1% (MD = 11.24, 95%Cl (6.87, 15.61), *p* < 0.00001), TLC% (MD = 5.28, 95%Cl (0.69, 9.87), *p* = 0.02)], but no significant difference in the post-treatment DLCO% [(MD = 4.40, 95%Cl (−2.29, 11.09), *p* = 0.20)]. Moreover, the data showed that TwHF combined with conventional treatment significantly reduced the HRCT integral of patients [MD = -0.65, 95% (-1.01, -0.30), *p =* 0.0003], the level of erythrocyte sedimentation rate (MD = −9.52, 95%Cl (−11.55, −7.49), *p* < 0.00001), c-reactive protein (CRP) (MD = −8.42, 95%Cl (−12.47, −4.38), *p* < 0.0001), and rheumatoid factor (MD = −25.48, 95%Cl (−29.36, −21.60), *p* < 0.00001). Compared to conventional therapy, TwHF combined with conventional therapy significantly improved clinical effects (RR = 1.33, 95%Cl (1.17, 1.51), *p* < 0.0001), in five trials with 354 patients. In terms of improvement of symptoms and signs, the TwHF group showed a more significant improvement than the conventional treatment group (Cough (MD = −0.96, 95%Cl (−1.43, −0.50), *p* < 0.0001), velcro rales (MD = −0.32, 95%Cl (−0.44, −0.20), *p* < 0.00001), shortness of breath (MD = −1.11, 95%Cl (−1.67, −0.56), *p* < 0.0001)], but no statistical difference in dyspnea (MD = −0.66, 95%Cl (−1.35, 0.03), *p* = 0.06). There was no statistical significance in the incidence of adverse reactions.

**Conclusion:** The performed meta-analysis indicated that TwHF combined with conventional treatment was more beneficial to patients for improving symptoms, lung function and laboratory indicators. As it included studies with relatively small sample size, the findings require confirmation by further rigorously well-designed RCTs.

## Introduction

Connective tissue disease (CTD) is a group of autoimmune diseases characterized by the damage of connective tissue components in various parts of the body (Zhang and Kang, 2009). Multiple organs and systems are involved, and the sites of related pulmonary lesions include respiratory tract, stroma, alveoli, blood vessels, pleura, and diaphragm (Zhao et al., 2018). Connective tissue disease-associated interstitial lung disease (CTD-ILD) can occur in a variety of connective tissue diseases, such as rheumatoid arthritis (RA), primary Sjogren’s syndrome (pSS), systemic sclerosis (SSc), and so on. According to a population-based cohort study by Ng KH et al. (Ng et al., 2020), the risk of ILD in patients with the above connective tissue diseases is significantly increased, but the prevalence rates vary due to different detection methods. Different CTD-ILD can exhibit different types of clinical manifestations, imaging and pathological features (Rheumatology and Immunology branch of Chinese Medical Doctor Association, 2018), which presents different development abnormalities, leading to difficulties in early diagnosis and treatment. Clinically, it often requires respiratory, rheumatology, radiology and other disciplines to participate in the disease assessment.

One study showed that approximately forty percent of patients with ILD have associated CTD at the same time (Mira-Avendano et al., 2019). Some ILD patients can develop progressive pulmonary fibrosis, impeded lung function and, eventually, even respiratory failure (Fischer and Distler, 2019). CTD-ILD is now seriously affecting the quality of life in those patients, even life-threatening. Therefore, the early diagnosis of CTD-ILD patients, and select the most appropriate individualized treatment and formulate the corresponding follow-up plan according to the condition of the patients, so as to achieve the remission of CTD-ILD patients and the long-term stability of lung function, prolong the survival time of patients to the greatest extent, and improve the quality of life, are the urgent problems to be solved.

At present, the etiology and pathogenesis of CTD-ILD are still unclear, but immune-mediated pulmonary inflammation and subsequent fibrosis are key elements in the development of the condition (Atzeni et al., 2018). Therefore, glucocorticoid and immunosuppressive therapy are an essential choice of CTD-ILD (Mathai and Danoff, 2016). They can effectively prevent the progression and deterioration of ILD, and help maintain adequate lung function. For CTD-ILD patients with short course of disease, rapid progress and severe sympoms intensity, high-dose methylprednisolone shock therapy can be used at the beginning of treatment (Rheumatology and Immunology branch of Chinese Medical Doctor Association, 2018). Immunosuppressive drugs commonly used in clinical practice include cyclophosphamide, mycophenolate, azathioprine and tacrolimus (Jee and Corte, 2019). Moreover, the occurrence of adverse events requires further monitoring in multicenter trials. In addition, relevant studies about biology agent or antifibrotic drugs have shown a good efficacy and safety in the treatment of CTD-ILD, especially in NSIP mode (Duarte et al., 2019; Moran-Mendoza et al., 2019; Maher et al., 2020). The cost of treatment with those, however, is extremely high, leading to CTD-ILD patients being unable to follow up with the treatment for prolonged periods of time.

As a complementary and alternative therapy for CTD-ILD, Chinese herbal medicine has the advantages of being multi-targeting and safe, therefore it has gradually become a popular choice for managing the condition by patients. Our previous study found that certain traditional Chinese medicine compounds can interfere with the pulmonary fibrosis process by inhibiting the aging of fibroblasts, showing a good anti-fibrosis effect (Feng et al., 2019; Peng et al., 2021). Tripterygium wilfordii Hook. F (TwHF) is an important herb in traditional Chinese medicine. Data increasingly has been showing that the related extracts of TwHF have immunomodulatory, anti-inflammatory and anti-allergic effects, and is widely used for the treatment of rheumatoid arthritis in China (Bao and Dai, 2011; Bai et al., 2020; Hu et al., 2019). The results of mutiple clinical trials have demonstrated that TwHF monotherapy exhibits good efficacy in patients with active RA (Lv et al., 2015), SLE (Liu et al., 2014; Chen et al., 2020a), ankylosing spondylitis (Li et al., 2015; Ji et al., 2015), kidney disease (Ge et al., 2013; Wang, 2018)and some skin diseases, such as psoriasis (Lv et al., 2018). Zhang et al. found that TwHF can disrupt the process of CTD-ILD through multiple targets and multiple pathways (Zhang et al., 2021). In another study (Yang et al., 2011), TwHF inhibited the excessive apoptosis of pulmonary epithelial cells and the accumulation of extracellular matrix (ECM) in lung tissue by regulating the expression of immune/inflammatory regulatory factors, thus preventing the progress of pulmonary fibrosis. Furthermore, studies have shown that the therapeutic effect of TwHF is related to the regulation of the proportion between CD4+and CD8+T cells, the immune balance of Th17 cells and Tregs, and the differentiation of dendritic cells (Chen et al., 2015; Luo et al., 2019).

There is a plethora of anecdotal evidence of TwHF use in traditional Chinese medicine as an immunosuppressant with an outstanding curative effect. However, there is no reliable evidence-based medical research that confirms its efficacy in the treatment of CTD-ILD. In order to fully prove its effectiveness and safety, it is necessary to evaluate its preparation clinically. Therefore, we conducted a systematic review and meta-analysis following the PRISMA checklist in order to comprehensively evaluate all relevant clinical randomized controlled trials and provide more consistent scientific evidence for the clinical application of TwHF for CTD-ILD patients.

## Methods

This systematic review and meta-analysis was performed and reported according to the Preferred Reporting Items for Systematic Reviews and Meta-Analyses (PRISMA) guidelines and readers can access the protocol of this systematic review in International Prospective Register of Systematic Reviews (PROSPERO) (CRD42020210690).

### Literature Search Strategy

A comprehensive literature search was performed in the following electronic databases: PubMed, Embase, Cochrane Library, Web of Science, PsycINFO, Scopus, CNKI, WanFang, VIP and CBM database. All of the databases were searched to identify the relevant human clinical studies published until May 2021. Two reviewers (Yehui Li and Wen Zhu) conducted the literature search independently, the search strategy used was as follows: [(“*Tripterygium wilfordii* Hook F”) OR (“Tripterygium wilfordii”) OR (“TwHF”)] AND [(“Connective tissue disease”) OR (“CTD”) OR (“Rheumatoid arthritis”) OR (“Sjogren’s syndrome”) OR (“Systemic lupus erythematosus”) OR (“Systemic sclerosis”) OR (“Dermatomyositis”)] AND [(“Interstitial lung disease”) OR (“ILD”)].

### Inclusion Criteria

We believed that the research included in the meta-analysis should meet the following criteria: 1) The study design was confined to RCT regardless of blinding. 2) According to the classification and diagnostic criteria of RA, SLE formulated by the American College of Rheumatology (Andonopoulos et al., 1987; Hochberg, 1997; Aletaha et al., 2010), Primary Sjogren syndrome formulated by the American-European Consensus Group (Vitali et al., 2002) and the diagnostic criteria for interstitial lung disease formulated by the Thoracic Association (Raghu et al., 2011). 3) The studies provided the experimental group with TwHF in combination with conventional therapy while the control group with conventional therapy alone. Conventional therapy of CTD-ILD mostly consisted of the suppression of inflammation with corticosteroid or immunosuppressive therapy including azathioprine, ciclosporin, cyclophosphamide, mycophenolate mofetil or tacrolimus (Wells and Denton, 2014).

### Exclusion Criteria

Relevant clinical trials were manually removed if any of the following factors were identified: 1) duplicated articles, 2) inappropriate interventions, 3) incomplete data, 4) irrelevance to outcome indicators.

### Outcome Measures

Primary outcome measures included pulmonary function related indicators such as forced expiratory rate of the 1st second (FEV1%), forced expiratory volume (FVC%), total lung volume (TLC%), carbon monoxide diffusing capacity (DLCO%); HRCT integral (Austin et al., 1996), commended by The Outcome Measures in Rheumatology (OMERACT) (Saketkoo et al., 2014).

Secondary outcome measures included clinical efficacy, dyspnea, cough, shortness of breath and Velcro rales, according to the efficacy standards of “CTD-ILD” issued by the Chinese Medical Doctor Association (Rheumatology and Immunology branch of Chinese Medical Doctor Association, 2018), and laboratory indicators included c-reactive protein (CRP), erythrocyte sedimentation rate (ESR), rheumatoid factor (RF) are also evaluated.

Safety outcome measures included occurrence of adverse events.

### Data Extraction and Quality Assessment

Two reviewers (YL and WZ) independently searched, screened and selected the articles, then extracted, and examined all the data. The extracted data included: 1) basic information such as the name of lead author, publication year, and gender; 2) number of participants in total and in each group (experimental and control), and the average age; 3) details of interventions, treatments of control groups, and treatment duration; 4) outcomes from each study and adverse reactions. The two researchers (YL and WZ) also evaluated the methodological quality of all the RCTs based on the criteria in the Cochrane evaluation handbook of RCTs 5.1.0.

In terms of bias, the articles were divided into three grades: low risk, high risk and unclear risk according to the following quality items: randomization generation, allocation concealment, blinding of subjects, outcome assessment, incomplete outcome data and selective outcome reporting. In case of differing opinions, consensus was reached through consultation and discussion.

### Statistical Analysis

According to the RevMan 5.3 software (Cochrane Collaboration), risk ratios (RR) with 95% confidence intervals (CI) for dichotomous data and mean differences (MD) with 95% CIs for continuous data were reported. Heterogeneity was evaluated statistically using the *I* ^*2*^statistic. Meta-analysis was carried out using a random effects model if *I* ^*2*^ *>*50%*,* otherwise if *I* ^*2*^<50%, the fixed effects model was selected. A sensitivity analysis of HRCT integral, laboratory indicators including CRP, ESR, RF and improvement of dyspnea, cough, shortness of breath and Velcro rales was performed by deleting each study in sequence. Then, meta-analysis was re-conducted for the remaining studies.

## Results

### Literature Search Results

**FIGURE 1:**
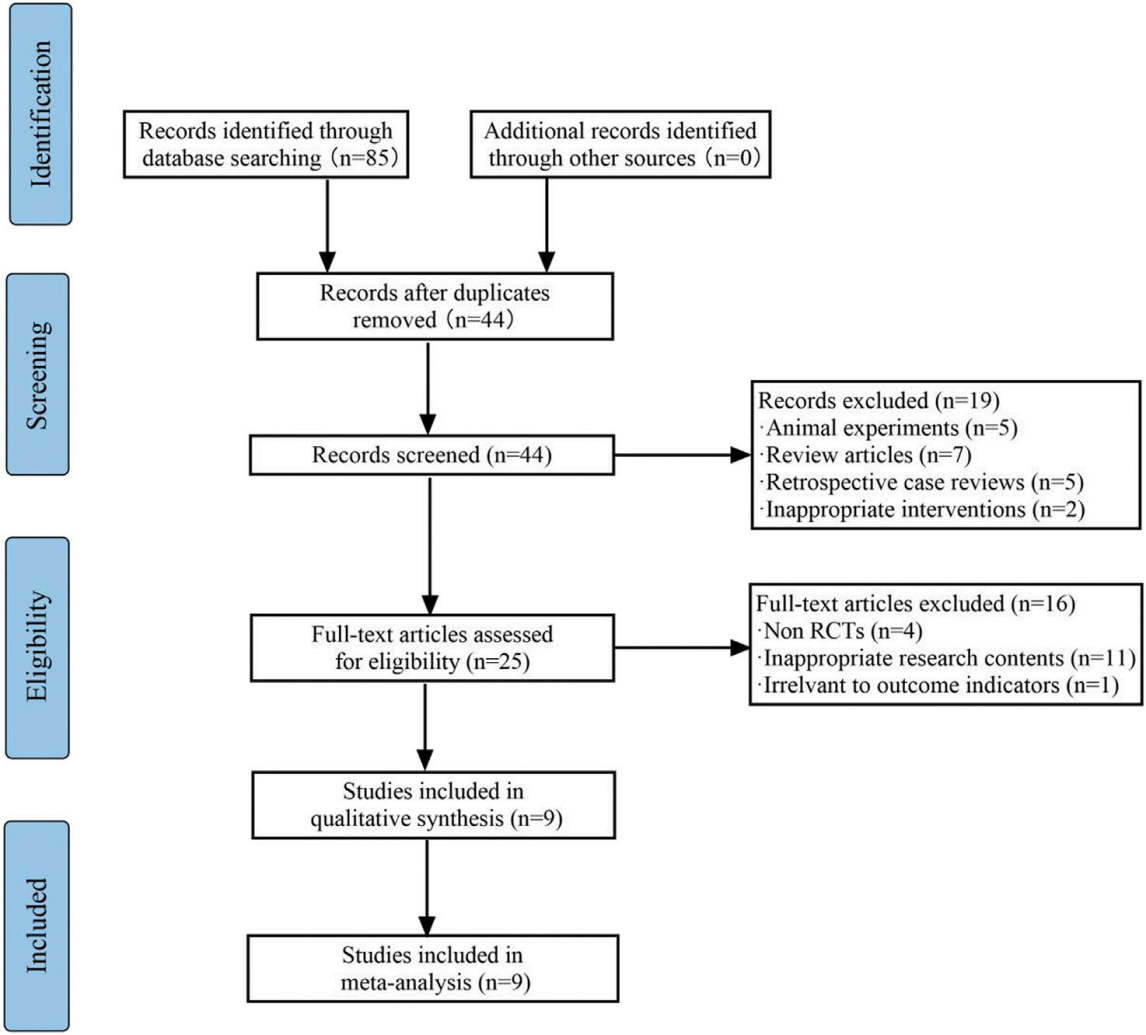
PRISMA flow diagram of the literature search process.

Based on the retrieval strategy, a total of 112 clinical studies were obtained. After screening according to the inclusion/exclusion criteria, nine articles were determined for further analysis (Figure 1).

### Description of Studies

There were nine RCTs included in the meta-analysis, involving 650 patients with CTD-ILD (Hu, 2015; Li et al., 2017; Yang et al., 2017; Lin et al., 2017; Dong, 2017; Gao, 2020; Chen et al., 2020a; Fan and Bai Ma, 2020; Chen et al., 2020b). All of the studies were conducted in China and the year of publication was between 2015 and 2021. There were 324 cases in the experimental group and 326 cases in the control group. Characteristics of the included studies included lead author, publication year, sample size, average age, interventions, outcome indicators, period of treatment and adverse reactions were summarized in Table 1.

**TABLE 1:** The characteristics of the included trials.

Study	No	Gender (M/F)		Average age(Y)		Interventions		Duration(M) T/C	Outcomes
	T/C	T	C	T	C	T	C	T/C	
Gao (2020)	25/30	16/9	19/11	52.95 ± 6.32	52.82 ± 6.41	TwHF + CTX	CTX	12/12	②⑦⑧⑪⑫⑬
Chen et al. (2020a)	50/50	22/28	20/30	45.42 ± 9.37	46.34 ± 9.65	TwHF + PAT	PAT	6/6	①②③④⑦⑧⑨⑪⑫⑬
Fan et al. (2020)	30/30	16/14	17/13	54.37 ± 3.52	55.18 ± 3.37	TwHF + MP	MP	6/6	①②⑧⑨⑩
Chen et al. (2020b)	20/20	7/13	8/12	53.58 ± 2.06	52.97 ± 1.05	TwHF + PAT	PAT	6/6	①⑤⑥
Li et al. (2017)	41/39	16/25	16/23	57.7 ± 10.3	56.9 ± 10.5	TwHF + CTX	CTX	6/6	①②③④⑤⑪⑫⑬
Yang et al. (2017)	30/30	2/28	2/28	50.3 ± 7.7	48.4 ± 8.1	TwHF + PAT	CTX + PAT	6/6	②⑥
Lin et al. (2017)	37/37	12/25	10/27	57.4 ± 7.5	56.3 ± 6.9	TwHF + PAT	PAT	6/6	①②⑤⑥⑧⑨⑩
Hu (2015)	31/30	17/44		56		TwHF + PAT + CTX	PAT + CTX	6/6	②⑦⑧
Dong (2017)	60/60	22/38	20/40	50 ± 3.43	51 ± 2.78	TwHF + DXM + CTX	DXM + CTX	7/7	⑦⑧

No., number of participants; T, treatment; C, control; M, male; F, female; Y, year; M, month; TwHF, Tripterygium wilfordii Hook. F; CTX, cyclophosphamide; DXM, dexamethasone; MP, methylprednisolone; PAT, prednisone acetate tablets. ①Clinical efficacy; ②HRCT score; ③FEV1%; ④FVC%,; ⑤TLC%,; ⑥DLCO%; ⑦improvement of dyspnea; ⑧improvement of cough; ⑨improvement of shortness of breath; ⑩improvement of Velcro rales; ⑪ CRP, ⑫ ESR, ⑬ RF.

### Description of Interventions

The control group was given glucocorticoid or immunosuppressive therapy. In addition to these conventional treatments in the control group, the experimental group was supplemented with TwHF.

### Quality Evaluation of Literature

**FIGURE 2:**
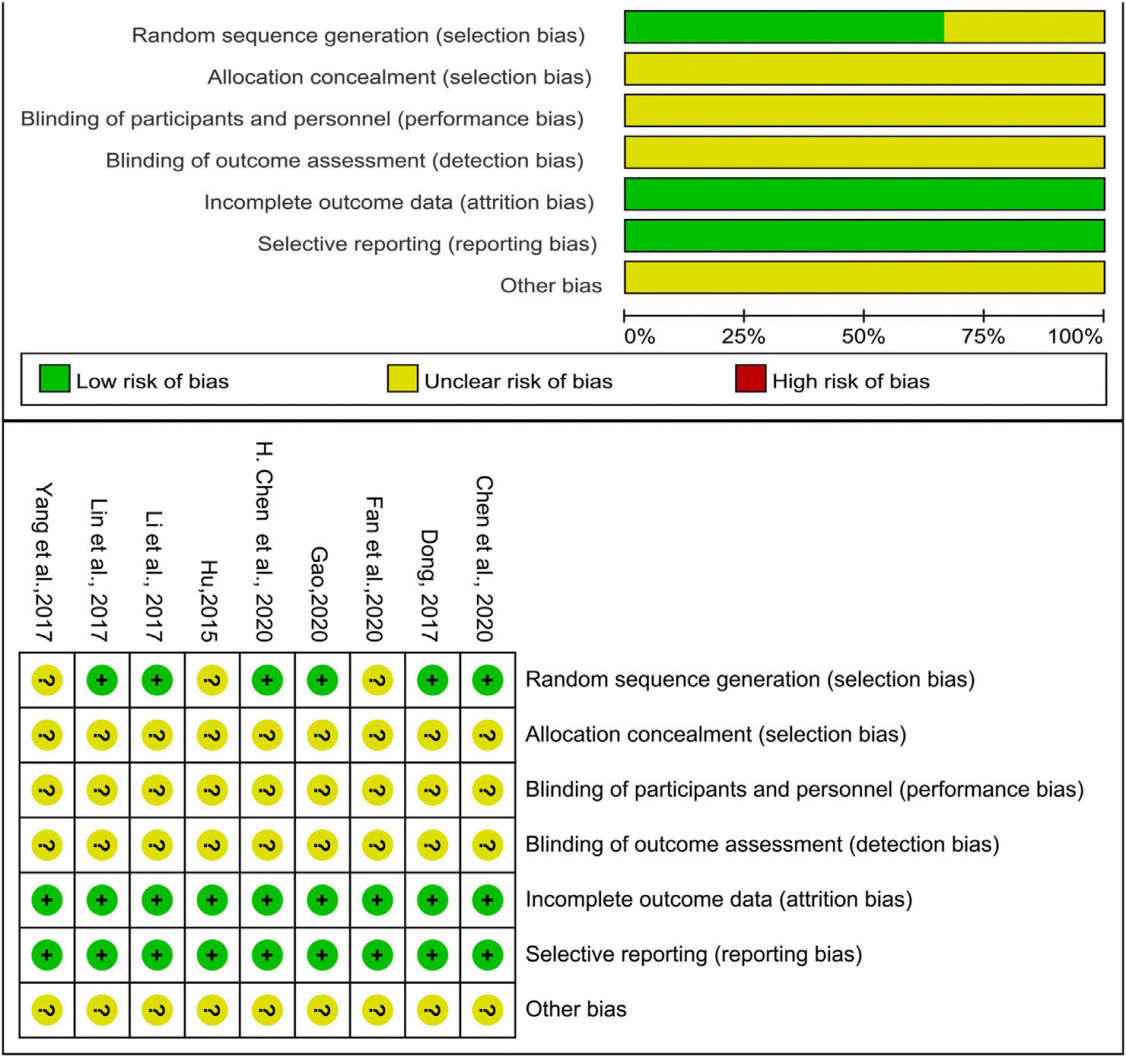
**|** Risk of bias summary and risk of bias graph.

All of the included trials mentioned applying randomization methodology. Six studies (Li et al., 2017; Lin et al., 2017; Dong, 2017; Chen et al., 2020a; Gao, 2020; Chen et al., 2020b) mentioned specific randomization grouping methods. Four of them used random number table to generate a sequence (Li et al., 2017; Chen et al., 2020c; Gao, 2020; Chen et al., 2020a), one used a lottery method to group participants (Lin et al., 2017), and the other used dynamic randomization method (Dong, 2017). None of the trials specified the methods of allocation concealment and the blinding procedures. This indicated that there were unclear risks of bias. No trials mentioned selective reporting, so a low risk of bias was chosen. Other biases were not determined. Figure 2 shows detailed information about the studies’ research methods quality.

### Outcome Measures

#### Primary Outcomes

##### Changes in Lung Function

**FIGURE 3:**
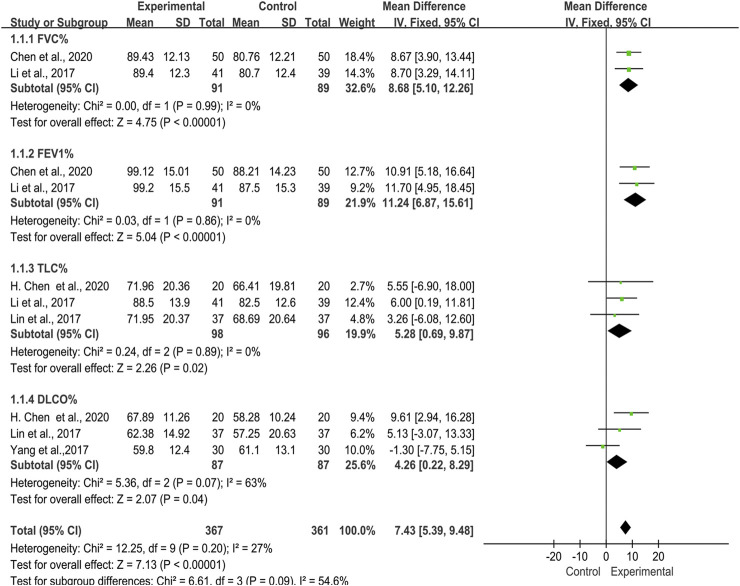
Forest plot of improvement of lung function with TwHF combined with conventional treatment versus pure conventional treatment.

Lung function data was included in five trials (Li et al., 2017; Lin et al., 2017; Yang et al., 2017; Chen et al., 2020a; Chen et al., 2020b), which included 354 patients in total (Figure 3). As the heterogeneity test showed of DLCO% was high (*I* ^*2*^ = 63%, *p* = 0.07), a random-effects model was applied to calculate the MD and 95%Cl so as to ensure reliability of the results. The results demonstrated the TwHF group, compared with the control group could improve patients’ lung function significantly (MD = 7.30, 95% (4.86, 9.74), *p* < 0.00001). Next, we separately described the lung function indexes such as FEV1%, FVC%, TLC% and DLCO%.

##### FEV1% and FVC%

Two trials (Li et al., 2017; Chen et al., 2020c), 180 patients, described changes of lung function in FEV1% and FVC%. The studies showed no significant heterogeneity in these two indicators [FEV1% (*I* ^*2*^ = 0%, *p* = 0.86); FVC% (*I* ^*2*^ = 0%, *p* = 0.99)]. The performed meta-analysis showed that there was a statistically significant difference between two groups in FEV1% [MD = 11.24, 95% (6.87, 15.61), *p* < 0.00001] and FVC% [MD = 8.68, 95% (5.10, 12.26), *p* < 0.00001], indicating that TwHF plus conventional treatment had advantages in improving FEV1% and FVC% compared with using conventional treatment alone.

##### TLC%

Three trials (Li et al., 2017; Lin et al., 2017; Chen et al., 2020a) reported 194 patients with TLC%. We noted no significant heterogeneity in two studies (*I* ^*2*^ = 0%, *p* = 0.89). According to the statistical difference of the results in TLC% [MD = 5.28, 95% (0.69, 9.87), *p* = 0.02], we concluded that the TwHF group could improve the TLC% of patients.

##### DLCO%

There were also three trials (Lin et al., 2017; Yang et al., 2017; Chen et al., 2020b) introduced DLCO%, which included 174 patients. The performed meta-analysis showed that the heterogeneity test was high (*I* ^*2*^ = 63%, *p* = 0.07), and there was no statistical significant difference between the TwHF group and control group [MD = 4.40, 95% (-2.29, 11.09), *p =* 0.20].

##### HRCT Integral Evaluation

**FIGURE 4:**
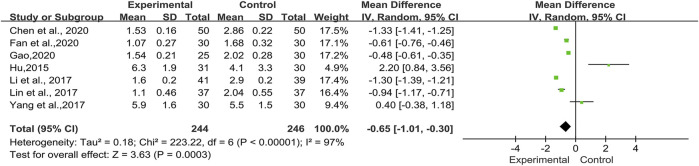
Forest plot of HRCT integral with TwHF combined with conventional treatment versus pure conventional treatment.

In nine trials, seven trials (Hu, 2015; Li et al., 2017; Lin et al., 2017; Yang et al., 2017; Chen et al., 2020c; Fan and Bai Ma, 2020; Gao, 2020), including 490 cases, reported HRCT integral (Figure 4). The heterogeneity was high (*I2* = 97%, *p* < 0.00001) and we applied the random-effects model in this meta-analysis. In the meta-analysis, a statistically difference [MD = -0.65, 95% (-1.01, -0.30), *p =* 0.0003] existed between TwHF combination group and control group, signifying that the combination of TwHF and conventional therapy could significantly reduce the HRCT integral of patients.

### Secondary Outcomes

#### Clinical Efficacy Evaluation

**FIGURE 5:**
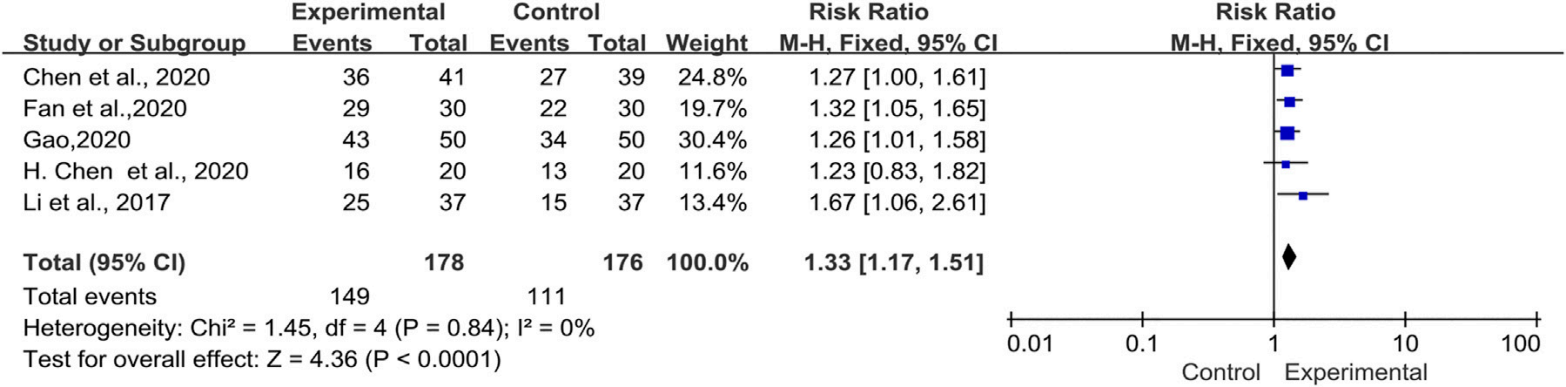
Forest plot of clinical efficacy with TwHF combined with conventional treatment versus pure conventional treatment.

Efficiency was evaluated based on interventions and controls in five trials (Li et al., 2017; Chen et al., 2020a; Chen et al., 2020b; Fan and Bai Ma, 2020; Gao, 2020) including 354 patients (Figure 5). The pooled results indicated that there was no significant heterogeneity (*I* ^*2*^ = 0%, *p* = 0.84). We chose the fixed-effects model for the analysis. The results showed that compared with western medicine alone, TwHF combined with conventional treatment can significantly improve the clinical efficiency of CTD-ILD patients [RR = 1.33, 95% (1.17, 1.51), *p* < 0.0001].

#### Assessment of Symptoms and Signs

**FIGURE 6:**
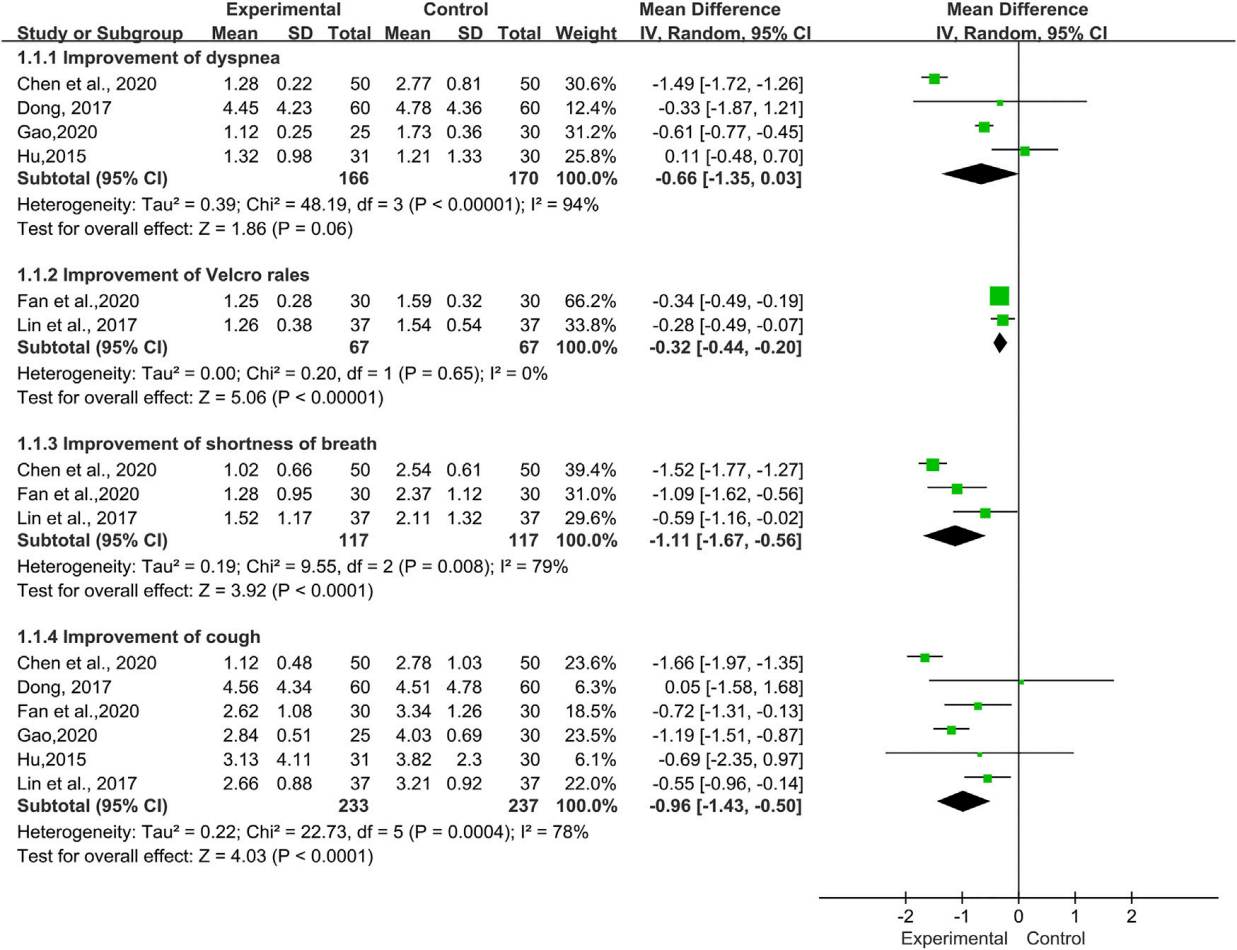
Forest plot of improvement of symptoms and signs with TwHF combined with conventional treatment versus pure conventional treatment.

A total of six trials (Hu, 2015; Dong, 2017; Lin et al., 2017; Chen et al., 2020c; Fan and Bai Ma, 2020; Gao, 2020) involving 150 patients reported improvement of various symptoms and signs. We applied a random-effects model according to the heterogeneity test results. (Figure 6).

#### Dyspnea

The improvement of dyspnea was reported in four trials (Hu, 2015; Dong, 2017; Chen et al., 2020b; Gao, 2020), which included 336 patients. High heterogeneity (*I* ^*2*^ = 94%, *p* < 0.00001) between the included studies was observed. The meta-analysis showed that there was no statistical significance between the TwHF group and the control group [MD = −0.66, 95% (−1.35, 0.03), *p =* 0.06].

#### Cough

Six trials (Hu, 2015; Dong, 2017; Lin et al., 2017; Chen et al., 2020a; Fan and Bai Ma, 2020; Gao, 2020), involving 470 patients, reported the improvement of cough. The data showed a high heterogeneity (*I* ^*2*^ = 78%, *p* = 0.0004) and a significant difference in the post-treatment of cough values favoring TwHF [MD = −0.96, 95% (−1.43, −0.50), *p* < 0.0001].

#### Shortness of Breath

Three trials (Lin et al., 2017; Chen et al., 2020b; Fan and Bai Ma, 2020) including 234 patients, reported improvement of shortness of breath. We noted a high heterogeneity (*I* ^*2*^ = 79%, *p* = 0.008) in the meta-analysis. The results were statistically significant, indicating that in the TwHF group the symptoms of shortness of breath were significantly improved compared with the control group [MD = −1.11, 95% (−1.67, −0.56), *p* < 0.0001].

#### Velcro Rales

Fan et al., 2020 and Lin et al., 2017 reported Velcro rales in 134 patients, which had no significant heterogeneity (*I* ^*2*^ = 0%, *p* = 0.65). The pooled results indicated that there was a significant statistical difference between two groups (MD = −0.32, 95% (−0.44, -0.20), *p* < 0.00001), indicating that Velcro rales in the combined group were more likely to improve in clinical practice.

#### Laboratory Indicators

**FIGURE 7:**
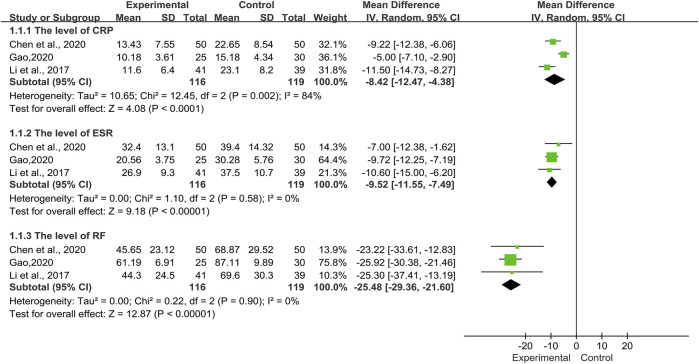
Forest plot of the level of laboratory indicators with TwHF combined with conventional treatment versus pure conventional treatment.

Three trials (Li et al., 2017; Chen et al., 2020c; Gao, 2020) involving 235 patients, compared the TwHF group with a pure conventional treatment in the post-treatment of laboratory indicators including CRP, ESR, and RF. As illustrated in Figure 7, the results showed that there was no significant heterogeneity, aside from CRP (*I* ^*2*^ = 84%, *p* = 0.002). Thus, a random-effects model was applied for analysis. According to the statistical difference of the CRP [MD = −8.42, 95%Cl (−12.47, −4.38), *p* < 0.0001], ESR [MD = −9.52, 95%Cl (−11.55, −7.49), *p* < 0.00001], and RF [MD = −25.48, 95%Cl (−29.36, −21.60), *p* < 0.00001], we thought that TwHF combined with conventional therapy could effectively reduce the level of laboratory indicators such as CRP, ESR, and RF.

#### Adverse Events

**FIGURE 8:**
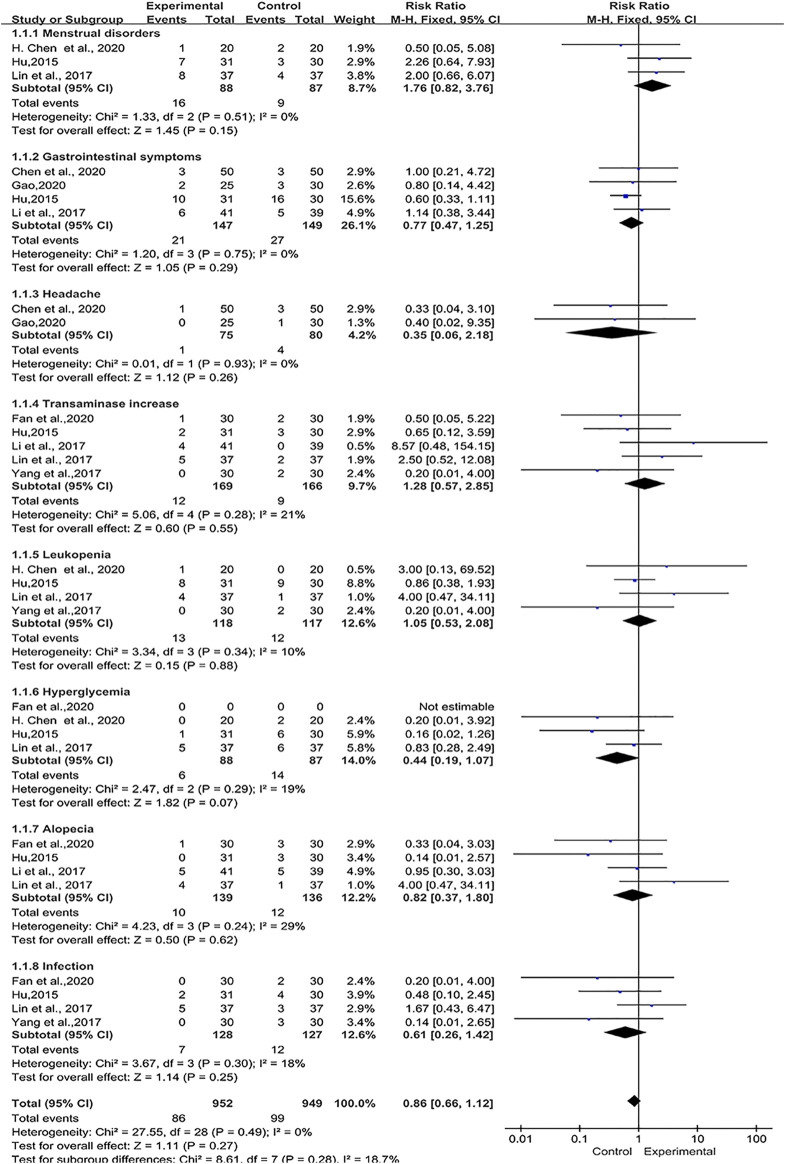
Forest plot of adverse events with TwHF combined with conventional treatment versus pure conventional treatment.

Eight trials reported AEs (Hu, 2015; Li et al., 2017; Yang et al., 2017; Lin et al., 2017; Dong, 2017; Gao, 2020; Chen et al., 2020a; Chen et al., 2020b; Fan and Bai Ma, 2020). AE details are shown in Table 2 These studies clearly reported the accurate numbers of different symptoms of AEs including menstrual disorders, gastrointestinal symptoms, headache, transaminase increase, leukopenia, hyperglycemia, alopecia, and infection in TwHF group and control group. Furthermore, only one trial reported Cushing’s syndrome alone, so a meta-analysis of this adverse event was not available (Hu, 2015). The pooled results showed that there was no statistically significant difference in the incidence of AEs between the TwHF combined treatment group and the conventional treatment group (Figure 8). However, the relationships between the AEs and the interventions were not further discussed in any study.

**TABLE 2:** Adverse events reported in the studies.

Study	Adverse events	
	Intervention	Control
Fan et al. (2020)	One alopecia; no hyperglycemia and infection; one transaminase increased	Three alopecia; two hyperglycemia, infections and transaminase increased
Chen et al. (2020a)	Three gastrointestinal symptoms; one headache	Three gastrointestinal symptoms and headache
Gao (2020)	Two gastrointestinal symptoms	Three gastrointestinal symptoms; one headache
Chen et al. (2020b)	One menstrual disorder and leukopenia; no hyperglycemia	Two menstrual disorders and hyperglycemia; no leukopenia
Li et al. (2017)	Five alopecia; six gastrointestinal symptoms; three mouth ulcers	Five alopecia and gastrointestinal symptoms; four mouth ulcers
Lin et al. (2017)	Four alopecia; eight menstrual disorders; five hyperglycemia, infections and transaminase increased; four leukopenia	One alopecia; four menstrual disorders; six hyperglycemia; three infections; two transaminase increased; one leukopenia
Yang et al. (2017)	No adverse events	Three infections and thrombocytopenia; two leukopenia and transaminase increased; one gastrointestinal symptoms
Hu (2015)	Five cushing syndrome; seven menstrual disorders; one hyperglycemia; no alopecia; ten gastrointestinal symptoms; two transaminase increased and infections; eight leukopenia	Nineteen cushing syndrome; three menstrual disorders; six hyperglycemia; three alopecia; sixteen gastrointestinal symptoms; three transaminase increased; four infections; nine leukopenia

*From:* Moher D, Liberati A, Tetzlaff J, Altman DG, The PRISMA Group (2009). Preferred Reporting Items for Systematic Reviews and Meta-Analyses: The PRISMA Statement. PLoS Med 6(7): e1000097. doi:10.1371/journal.pmed1000097.

For more information, visit: www.prisma-statement.org.

#### Sensitivity Analysis

**FIGURE 9:**
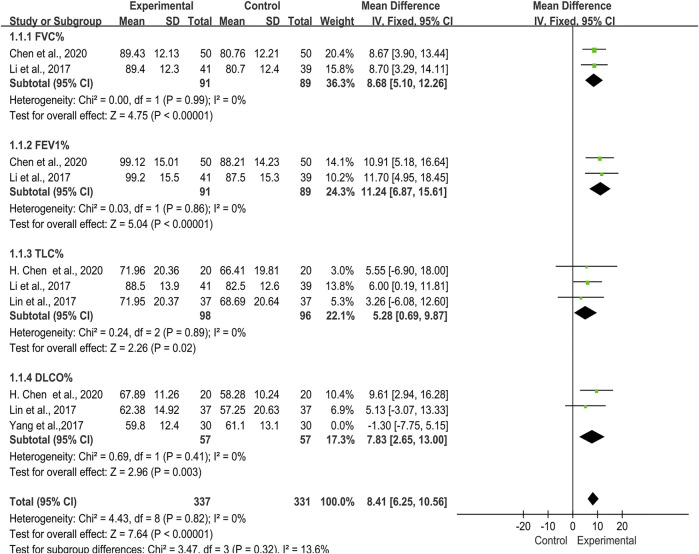
Forest plot of sensitivity analysis of lung function with TwHF combined with conventional treatment versus pure conventional treatment.

**FIGURE 10:**
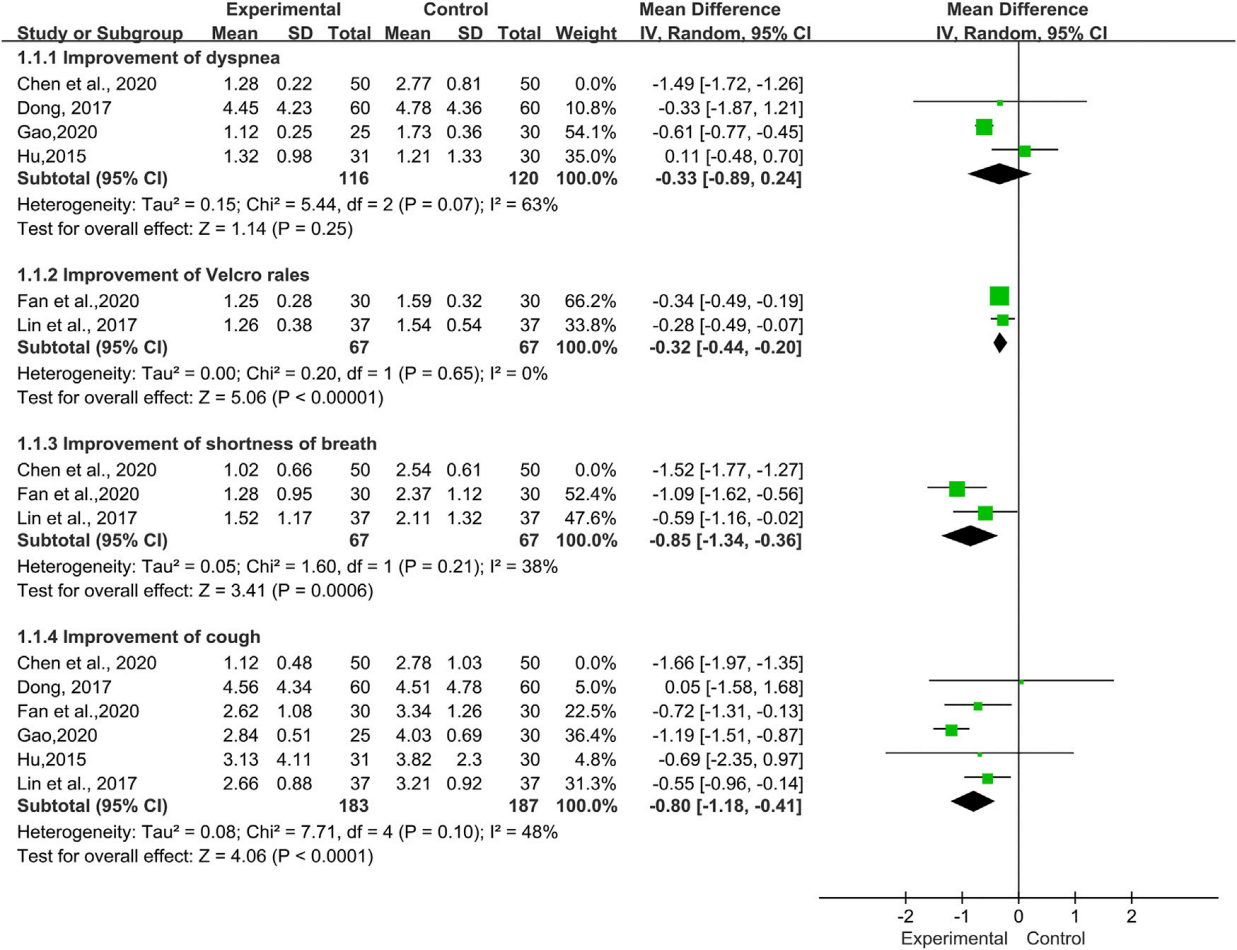
Forest plot of sensitivity analysis of symptoms and signs improvement with TwHF combined with conventional treatment versus pure conventional treatment.

**FIGURE 11:**
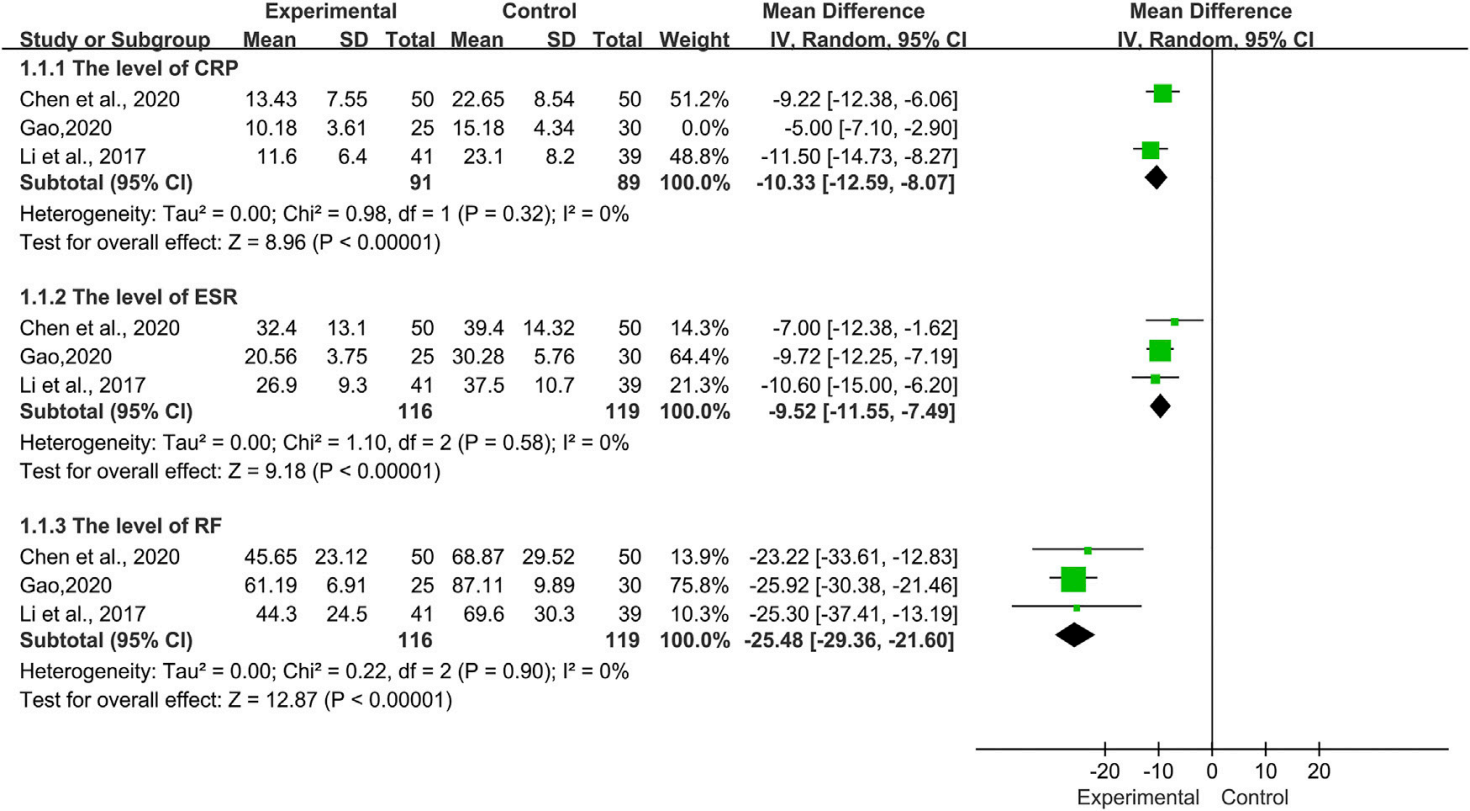
Forest plot of sensitivity analysis of laboratory indicators with TwHF combined with conventional treatment versus pure conventional treatment.

The results of all studies in the fixed-effects model showed good consistency. For the continuous data, we eliminated the included studies one by one, and the others were reanalyzed by meta-analysis. The results showed that HRCT integral did not change substantially, but in DLCO% index, after excluding Yang et al., 2017, the heterogeneity test decreased from 63 to 0%, *p* = 0.003 (Figure 9). The data suggested that Yang et al., 2017 was the main reason for the heterogeneity in DLCO%, which may be related to the blowing state of the patients participating in the study during the lung function examination. In terms of symptoms improvement, after excluding Chen et al., 2020a, the heterogeneity test of dyspnea improvement decreased from 94 to 63%, *p* = 0.25, the heterogeneity test of shortness of breath decreased from 79 to 38%, *p* = 0.0006, and the heterogeneity test of cough improvement decreased from 78 to 48%, *p* < 0.0001 (Figure 10). In addition, after the deletion of Gao, 2020, the heterogeneity test of CRP level decreased from 84 to 0%, *p* < 0.00001 (Figure 11). These findings suggested that Chen et al., 2020a and Gao, 2020 were the reasons for the heterogeneity of symptom improvement and CRP level results.

#### Analysis of Publication Bias

We planned to use the total effective rate as the outcome index for Funnel plots analysis of the included studies, but only five studies met the requirements. Therefore, no publication bias analysis was conducted.

## Discussion

Based on the current lack of high-level scientific evidence, the management and treatment of CTD-ILD patients are relying on case reports and clinical experience. It is worth noting that many disease remission anti-rheumatic drugs (DMARDs) for CTD treatment have different degrees of adverse reactions, including pulmonary infection, interstitial pneumonia and pulmonary sarcoidosis, of which interstitial pneumonia is the main type. The most common drugs involved include methotrexate and leflunomide (Skeoch et al., 2018; Gao and Moua, 2020). With the development of modern medicine, the treatment of CTD-ILD is diversified, but there is still a considerable number of patients with end-stage respiratory failure, prompting people to consider anti-fibrosis, hematopoietic stem cell transplantation, lung transplantation and other treatments (De Cruz and Ross, 2013; Crespo et al., 2016; Pradère et al., 2018). Therefore, new treatment methods or standardized therapeutic measures are urgently needed.

Recent research has increasingly been focusing on the role of alternative therapies such as traditional Chinese medicine for the treatment of rheumatic diseases. Tripterygium wilfordii Hook. F (TwHF) is a Chinese herb also known as Lei Gong Teng (thunder god vine). It has been found to contain more than 70 chemical components (Chen et al., 2018) and has been confirmed to have definite anti-inflammatory, immunomodulatory effects, in addition to being effective for improving the symptoms in many CTDs. For examble, Zhou YY et al. provided more scientific and convincing evidence for the treatment of rheumatoid arthritis by TwHF in a meta-analysis (Zhou et al., 2018). The main active components of TwHF can reduce capillary permeability, inhibit inflammatory cytokines or chemokines, and regulate the expression of various inflammatory mediators, thereby reducing lung injury (Law et al., 2011; Wei and Huang, 2014).

Triptolide improved the pulmonary function by inhibiting myofibroblast activation and collagen deposition in lung tissues. Triptolide also mitigated pulmonary fibrosis partly by downregulating nicotinamide adenine dinucleotide phosphateoxidase 2 (NOX2) through the NF-κB pathway (Yuan et al., 2019) and increase the alveolar space (Hoyle et al., 2010). TwHF may upregulate CD4+CD25+regulatory T cells and improve immunity, which has been found to be beneficial for the improvement of lung function in another study (Lei and Jian, 2012). However, there is no clinical evidence-based medicine summary of TwHF for the treatment of CTD-ILD.

Conducting a systematic review and meta-analysis of the efficacy and safety in the treatment of CTD-ILD utilised TwHF has never been attempted before. This study reviewd nine RCTs consisting of 650 patients. Based on the meta-analysis results, we found that TwHF combined with western medicine was superior to pure western medicine in terms of improving lung function, HRCT integral, velcro rales, cough, and shortness of breath. Moreover, as ESR and CRP are representative inflammatory indexes in many CTD, we also conducted corresponding analysis. As a result, TwHF could significantly reduce the level of CRP, ESR and other inflammatory factors indicators, with good clinical efficacy.

Nevertheless, adverse events are always a focus of concern. TwHF may be harmful to the liver, kidneys, reproductive tissues, and immune tissues. In this meta-analysis, eight studies (Hu, 2015; Li et al., 2017; Yang et al., 2017; Lin et al., 2017; Gao, 2020; Chen et al., 2020a; Fan and Bai Ma, 2020; Chen et al., 2020b) mentioned the occurrence of adverse events including menstrual disorders, gastrointestinal symptoms, headache, transaminase increase, leukopenia, hyperglycemia, alopecia, and infection, but none of them dropped out of the study. The diversity of the components makes it difficult to analyze the safety of TwHF extracts. Subgroup analysis was not performed due to the different preparations of Tripterygium wilfordii and the limited data. Through the analysis of adverse events, it showed that there was no statistical significance between the TwHF combined treatment group and the control group. Although this study’s findings showed that the adverse events caused by TwHF were not significantly different from those caused by immunosuppressive agents, there is a clear need for improving prevention and management of patients’ tolerance for TwHF. Once adverse events appear, the patient should discontinue medication immediately and the clinician should take steps to manage the adverse event if necessary.

There are a number of limitations of this study. Although we have searched the basic Chinese and English databases, there are few randomized controlled trials on TwHF in the treatment of CTD-ILD or ILD. In addition, 100% of the trials included in this study were conducted in China, which may be viewed as certain ethical bias. Furthermore, there are few included studies, small sample capacity and lack of long-term follow-up. Although all the nine included trials mentioned the randomized grouping method, only six of them mentioned a specific random grouping method, among which 4 trials used random number table to generate a sequence (Li et al., 2017; Chen et al., 2020b; Gao, 2020; Chen et al., 2020c), one used a lottery method to group (Lin et al., 2017), and the other used dynamic randomization method (Dong, 2017). None of the studies included in this meta-analysis included blinding or allocation information. Furthermore, the quality of literature was found to be low, which reduced the credibility and reliability of its findings. Besides, as the course of medication were not uniform across the studies, there were some differences in the use of immunosuppressive agents in various studies, which may be the reason for the heterogeneity of related indicators. However, the treatment duration and the choice of immunosuppressive agents largely depend on the severity of the disease and individual tolerance to drugs. According to the nine included trials, we noted that T. wilfordii tgpolyglycoside (TWP) was used in six trials (Li et al., 2017; Lin et al., 2017; Chen et al., 2020a; Gao, 2020; Chen et al., 2020a; Fan et al., 2020) and Glucosidorum Tripterygll Totorum (GTT) was used in three trials (Hu, 2015; Dong, 2017; Yang et al., 2017). Different kinds of Tripterygium preparations may produce a certain heterogeneity, thus influencing the results and reducing the robustness of the conclusion. As a result, the findings of this study only served as a reference for the clinical application of TwHF in the treatment of CTD-ILD. Based on the published data of TwHF in the treatment of CTD-ILD, it is difficult to judge its potential in the treatment of CTD-ILD. It is suggested that in the design of clinical studies in the future, we should choose objective, international and universal therapeutic indexes as far as possible. At the same time, it is important that large-scale, multicenter and high-quality randomized controlled trials are conducted. We are looking forward to stronger evidence to confirm or refute the results reported in this study.

## Conclusion

In conclusion, evidence of this paper was found to support the fact that TwHF combined with conventional therapy provided statistically significant and clinically important improvement in CTD-ILD. To further support the conclusion, stronger scientific evidence is needed.

## Data Availability

The original contributions presented in the study are included in the article/Supplementary Material, further inquiries can be directed to the corresponding authors.
